# Machine Learning Applications with Sensors for Indoor Air Quality Research

**DOI:** 10.3390/s26092909

**Published:** 2026-05-06

**Authors:** Cosmina-Mihaela Rosca, Adrian Stancu

**Affiliations:** 1 Department of Automatic Control, Computers, and Electronics, Faculty of Mechanical and Electrical Engineering, Petroleum-Gas University of Ploiesti, 39 Bucharest Avenue, 100680 Ploiesti, Romania; cosmina.rosca@upg-ploiesti.ro; 2 Department of Business Administration, Faculty of Economic Sciences, Petroleum-Gas University of Ploiesti, 39 Bucharest Avenue, 100680 Ploiesti, Romania

**Keywords:** air quality prediction, indoor air quality index, machine learning, sensor-based monitoring, time series modeling, smart buildings, public and private datasets

## Abstract

Nowadays, people spend over 80% of their lives indoors, which makes indoor air quality (IAQ) research important. The paper presents, firstly, a structured overview of publicly available IAQ datasets suitable for machine learning (ML) research, secondly, a comparative analysis of the reviewed datasets, thirdly, an ML-oriented mapping between tasks and algorithms, to outline the algorithmic families that are most appropriate given the dataset structure and the prediction target, and fourthly, an investigation on IAQ–ML using custom-made solutions that include sensors for data acquisition. The methodology included an analysis of 1162 papers from the Web of Science, 1536 from Scopus, and 756 from IEEE Xplore, between 1 January 2020 and 31 December 2025, to capture recent trends in ML-based IAQ research. The findings show that linear regression (132 articles), Logistic regression (91), random forest—RF (77), Long Short-Term Memory—LSTM (77), Principal Component Analysis (63), and Elastic Net are the most popular among researchers. Most studies report accuracy over 90%, with maximum values of 99.37% for LSTM and 99.20% for RF. In the case of regression, the R^2^ values range between 82% and 98%, especially for CO_2_ and PM_2.5_ prediction. eXtreme Gradient Boosting or hybrid RF-LSTM architectures achieve R^2^ values of up to 99%. The IAQ public and private datasets analyzed for this study provide a strong foundation for transfer learning, but differences require careful preprocessing to ensure consistent comparisons and reliable conclusions. The distribution of articles by sensor type for IAQ parameters shows that linear regression remains the most widely used ML method (26 studies), followed by LSTM (19) and RF (18). The research results confirm that there is no universal algorithm for IAQ, and the quality and structure of the data contribute to the success of ML models. This study aims to be a foundation for the development of future intelligent IAQ monitoring systems.

## 1. Introduction

Indoor air quality (IAQ) has become a major public health and building-management concern due to the large fraction of time people spend indoors in homes, offices, classrooms, and public facilities (93% in the US [1]). Exposure to indoor pollutants, such as particulate matter (PM_2.5_ and PM_10_), carbon dioxide (CO_2_), carbon monoxide (CO), volatile organic compounds (VOCs), nitrogen dioxide (NO_2_), ozone (O_3_), as well as biological aerosol, has been associated with respiratory and cardiovascular effects, reduced cognitive performance, and aggravated symptoms for sensitive populations. Unlike outdoor pollution, indoor exposure is strongly shaped by building characteristics (e.g., ventilation type, filtration, and airtightness), occupant behavior (e.g., cooking, cleaning, and window opening), and microclimatic factors (e.g., temperature and relative humidity—RH). IAQ monitoring is a context-dependent problem that is analyzed using multi-sensor time series, heterogeneous variables, missing data, and non-stationary pollutant sources.

Traditionally, IAQ is approached on fixed thresholds, deterministic physical models, or rule-based ventilation control strategies. This approach struggles to capture nonlinear relationships between environmental variables and pollutant dynamics. In recent years, machine learning (ML) has emerged as a complement to traditional approaches by forecasting, regression, classification, anomaly detection, sensor calibration, and indoor air quality index (IAQI) estimation. ML models leverage correlations between low-cost sensor readings and reference measurements, learn temporal dependencies in pollutant evolution, provide real-time predictions, inform about threshold exceedances, and operate in the microclimate of the intelligent building control. For example, regression models estimate PM concentrations from proxy variables, classification models identify pollution events or ventilation states, and sequence models forecast short-term CO_2_ levels to proactively manage ventilation.

The first barrier of this approach is the availability and cost of reliable sensing infrastructure. High-quality sensors, particularly for PM size fractions, NO_2_, O_3_, or speciated VOCs, can be expensive, and their maintenance and calibration require additional resources. As a result, researchers and practitioners increasingly rely on publicly available datasets collected in research buildings, smart homes, laboratories, or through citizen-sensing campaigns. Public datasets accelerate experimentation and reproducibility, but they also vary substantially in sampling rate, duration, sensor types, labeling quality, ground truth availability, spatial granularity, and metadata completeness. This heterogeneity complicates the selection of appropriate ML methods and prevents straightforward comparison across studies.

The IAQ–ML literature spans diverse tasks (e.g., pollutant concentration prediction, IAQI estimation, event detection, occupancy inference, and sensor drift compensation). Each task has different evaluation metrics. The algorithm choice is often influenced by the dataset. Without a structured mapping between dataset characteristics, modeling tasks, algorithm performance, a unified formalism, and quality data in the training stage, it is difficult for new researchers to identify a suitable starting point or to design meaningful baselines.

Motivated by these limitations, and adding the fact of the practical difficulty of collecting IAQ data using costly sensors, this paper provides a review centered on public datasets and ML methods for IAQ modeling. The work aims to support reproducible and accessible research by clarifying what data resources are available and what types of ML algorithms are best suited to them. This paper, therefore, emphasizes the relationship between (i) pollutant/environmental variables typically present in public IAQ datasets, (ii) the modeling tasks they enable, and (iii) the ML families that have demonstrated strong performance under comparable conditions.

The aim of this review is to analyze IAQ in the ML context, with focus on datasets that include indoor pollutant measurements and associated environmental variables (e.g., temperature and RH), and, where available, contextual information such as occupancy indicators, ventilation status, building metadata, or outdoor reference measurements. The review addresses ML algorithms. While deep learning (DL), transfer learning, and hybrid modeling are included where relevant, this review prioritizes clarity and comparability across datasets, highlighting the practical constraints that arise when applying ML to real-world IAQ data.

This paper makes four main contributions to the IAQ–ML research domain:

1. A structured overview of publicly available IAQ datasets suitable for ML research, with emphasis on their applicability to common IAQ tasks. For each dataset, we summarize key properties such as measured pollutants, environmental variables, sampling frequency, duration, location or context, labeling or ground truth availability, and literature-reported data-quality issues.
2. A comparative analysis of the reviewed datasets, using a consistent set of criteria relevant to ML practice.
3. An ML-oriented mapping between tasks and algorithms, consolidating evidence on which algorithmic families (e.g., linear models, support vector machines (SVM), random forest (RF), Gradient Boosting (GB), recurrent/temporal neural networks, and probabilistic approaches) are most appropriate given the dataset structure and the prediction target. We discuss strengths and limitations with respect to nonlinearity, temporal dependency, interpretability, computational cost, and robustness.
4. An investigation on IAQ–ML using custom-made solutions that include sensors for data acquisition.

This paper is structured as follows. Section 2 covers core IAQ concepts and reviews commonly studied pollutants, sensing approaches, and IAQ indicators reported in prior work. Section 3 presents the methodology of the paper, which is organized around three main axes. Section 4 describes the ML survey through the literature on IAQ, and Section 5 introduces the public datasets for IAQ. Section 6 examines private datasets, and Section 7 explores ML models related to data acquisition from sensors. Discussion and conclusions are described in Section 8 and Section 9, respectively.

## 2. Core IAQ Concepts

IAQ refers to the quality of the air breathed by the occupants of a building throughout their stay inside. Air quality directly affects the health of people who work indoors. Technically, IAQ is determined based on the concentrations of pollutants, which can be physical, chemical, and biological in nature, along with microclimatic conditions represented by temperature and RH. These parameters directly affect the health and well-being of occupants inside buildings when their values exceed certain thresholds specific to each monitored parameter [2].

Over time, researchers have been concerned with monitoring these parameters, as well as the necessary intervention strategies when they exceed threshold values. Table 1 summarizes the parameters evaluated for calculating the IAQ, their threshold, and the need to monitor the indicators. The red color corresponds to gaseous indicators, green equals suspended particles, blue matches microclimatic parameters, and yellow indicates auxiliary parameters from certain research or datasets.

Beyond listing measurable parameters, IAQ must be identified as a dynamic process influenced by pollutant generation, pollutant removal, and the way occupants perceive and physiologically respond to indoor air conditions. In most buildings, pollutant levels are not constant [3]. The pollutants fluctuate with occupancy patterns, outdoor air conditions, ventilation schedules, cleaning activities, and intermittent sources such as cooking, printing, or the use of solvents. As a result, IAQ assessment should consider both instantaneous exceedances (short-term peaks) and longer-term averages, because health effects can be linked to acute exposures (e.g., irritation caused by VOC spikes) as well as chronic exposure (e.g., long-term particulate matter) [4].

**Table 1:** IAQ parameters utility and their threshold [5,6].

Parameter	Utility	Threshold
Carbon dioxide (CO_2_)	Indicator of indoor ventilation	~400–999 ppm
Carbon monoxide (CO)	Toxic gas from combustion monitoring	0–8.7 ppm
Volatile Organic Compounds (VOC)	Indoor chemicals that cause irritation	1–249 mg/m^3^
Nitrogen dioxide (NO_2_)	Indicator of combustion and traffic-derived gases indoors	<100 ppb
Ozone (O_3_)	Identification of the reactive gas	0–0.06 ppm
Particulate matter 1 (PM_1_)	Ultrafine particles penetrate deep into the respiratory system	0–34 μg/m^3^
Particulate matter 2.5 (PM_2.5_)	Fine particles are responsible for respiratory and cardiovascular effects	0–50 μg/m^3^
Particulate matter 10 (PM_10_)	Larger particulates that affect general respiratory irritation	0–75 μg/m^3^
Temperature	Affects occupant comfort	~21–27 °C
Relative Humidity (RH)	Influences thermal comfort and sensor accuracy for particulate measurement	~40–60%
Light	The level of lighting inside the room	300–500 lux
Formaldehyde (CH_2_O)	Emission of household products	0–0.1 ppm
Sulfur Dioxide (SO_2_)	Indicator of combustion-related indoor pollution and irritation risk	<75 ppb
Ammonia (NH_3_)	Indicator of cleaning products, human activity, industrial sources, etc.	<25 ppm

Threshold values refer to reference ranges for an indoor environment considered acceptable in terms of air quality. These values come from international guidelines and standards. These do not constitute universal threshold values, as the recommended values vary slightly depending on the context. The first context can be considered the type of building (residential, educational, and industrial). The second context relates to the target population (adults, children, and sensitive individuals suffering from various conditions). The primary sources for the thresholds used are: World Health Organization (WHO) for PM_2.5_, PM_10_, NO_2_, O_3_, and CO [7,8], the U.S. Environmental Protection Agency (EPA) for CO, O_3_, and PM [9], the European directives (Directive 2008/50/EC on ambient air quality and cleaner air for Europe) [10], and American Society of Heating, Refrigerating and Air-Conditioning Engineers (ASHRAE) standards, i.e., ANSI/ASHRAE Standard 62.1-2025, Ventilation and Acceptable Indoor Air Quality and ANSI/ASHRAE Standard 62.2-2025 Ventilation and Acceptable Indoor Air Quality in Residential Buildings [11,12]. The value thresholds for CO_2_ (~400–999 ppm) have been identified in the specialized literature as indicators of ventilation considered adequate for a room. For this reason, the thresholds are suitable for toxic limits [13]. For parameters without international consensus (e.g., VOC and TVOC), the ranges indicate typical values reported in indoor monitoring studies [2,6]. The authors recommend consulting local guidelines for the operational application of these thresholds.

A useful conceptual separation is between sources, transport, and receptors. Sources include outdoor infiltration (traffic-related NO_2_, O_2_, and PM), indoor activities (cooking emissions, resuspension of settled dust, and smoking where applicable), building materials and furnishings (formaldehyde and other VOCs), and combustion appliances (CO and NO_2_). Transport is mainly governed by air exchange rates, airflow pathways, and mixing between zones. Finally, receptors are the occupants, whose susceptibility varies depending on age, pre-existing respiratory or cardiovascular conditions, and time spent indoors. This framework demonstrates why “good IAQ” cannot be reduced to a single sensor reading, and it depends on the context and exposure.

Ventilation is often the first-line control strategy and is closely linked to CO_2_ as a practical indicator of occupancy-related pollutant accumulation. However, CO_2_ itself is not the only concern. Rather, it signals whether the ventilation rate is adequate to dilute human bioeffluents and other co-emitted contaminants. Still, CO_2_-based approaches have limits: a room can have acceptable CO_2_ while experiencing elevated PM from cooking, or elevated VOCs from cleaning products [14].

Another core concept is the interaction between microclimate (temperature and RH) and pollutant behavior. RH influences the survival of certain microorganisms. At the same time, RH affects perceived comfort. Temperature impacts emission rates from materials (higher temperatures may increase VOC off-gassing) and changes how occupants experience air freshness and discomfort. Consequently, IAQ management typically integrates thermal comfort targets with air cleanliness targets rather than treating them separately. In practice, IAQ evaluation uses threshold values as operational guidance, but those thresholds should be interpreted carefully.

## 3. Methodology

The methodology is designed as a systematic analysis of the specialized literature. Analyses are also introduced for datasets that can be public or custom-made, based on specialized sensors for analyzing specific parameters. The analysis focuses on IAQ using ML methods. The necessity of this approach is justified from the perspective of correlating the characteristics of the available data with the types of modeling tasks in relation to the families of algorithms used in practice.

The selection of scientific papers was conducted within the Web of Science (WoS) platform and is justified by the platform’s rigorous nature in the fields of engineering, environmental science, applied computer science, and the technical domain as a whole. The searches were limited to the period between 1 January 2020 and 31 December 2025 to capture recent trends in ML-based IAQ research.

**Figure 1:**
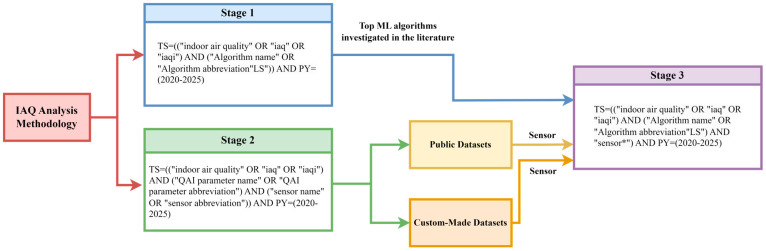
Paper research methodology (the asterisk in the word “sensor” was used to include all lexical derivatives).

Wos, Scopus, and IEEE Xplore databases were explored during the search phase. The final analytical framework focuses on the WoS dataset. This decision is justified by the higher consistency of indexing, reduced duplication, stronger alignment with high-impact journals in engineering and environmental sciences, and a large number of papers with open-access facility. The WoS dataset provided a curated corpus to support trend analysis without introducing additional heterogeneity across databases. A first set of WoS queries included specific IAQ terms and the names of ML algorithms, following the pattern indicated in Figure 1. A total of 175 ML algorithms were searched in the WoS database with year and IAQ semantic scope constraints. For example, for the RF algorithm, the WoS query was TS = ((“indoor air quality” OR “iaq” OR “iaqi”) AND (“random forest” OR “RF”)) AND PY = (2020–2025).

The identified works were filtered, retaining only studies that explicitly apply ML methods to IAQ data. All articles were retained in the results, regardless of whether they are for prediction or classification, and regardless of the target variable.

The methodological analysis of the literature was organized around three main axes. The first axis concerns the type of modeling task. Thus, in the first stage, prediction articles that perform regression tasks, as well as classification articles, are analyzed. The quality of the results presented in these papers is evaluated using specific metrics. The second axis focuses on the datasets used by the family of ML algorithms employed. Thus, the issue of datasets arose. These can be public or custom-made, built with the help of sensors. To support researchers, this paper identifies public datasets that can be used in the evaluation of ML algorithms. Furthermore, the question arose of identifying sensors that can measure values of specific IAQ parameters. The third axis focuses on an analysis of all articles in the literature that identify the IAQ domain according to six of the most studied ML algorithms and that include the concept of “sensor” in their keywords as part of a custom-made data acquisition. These results are analyzed in detail through a critical analysis.

**Figure 2:**
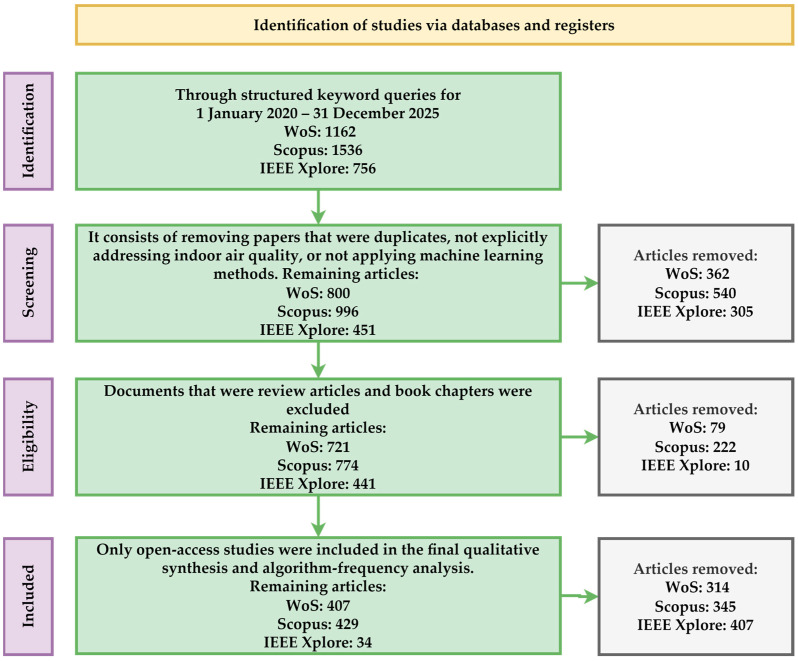
PRISMA flow diagram of study selection (2020–2025).

The process of selection and literature analysis followed a structure inspired by the PRISMA principles for identifying, selecting, and including studies [15], as shown in Figure 2.

The search strategy is based on systematic combinations of keywords that describe the field of IAQ and ML algorithms. The main terms used for IAQ were: “indoor air quality,” “iaq”, or “iaqi”. Multiple conditions generated based on main terms were combined using logical operators with the individual names of the ML algorithms. In total, 175 algorithms were considered. Each algorithm was queried individually in combination with the specific IAQ terms. The search was conducted in the WoS, Scopus, and IEEE Xplore databases. A total of 175 keyword combinations were executed independently in each database. The initial number of retrieved records was 1162 for WoS, 1536 for Scopus, and 756 for IEEE Xplore.

The screening and eligibility process followed the standard inclusion and exclusion criteria for PRISMA. Duplicate records generated by overlapping keyword combinations within the same database were identified and removed. After duplicate removal, the number of unique records was reduced to 800 for WoS, 996 for Scopus, and 451 for IEEE Xplore. After applying the exclusion criteria related to non-research articles, the number of records was reduced to 721 for WoS, 774 for Scopus, and 441 for IEEE Xplore.

The article selection process followed the PRISMA standard stages: identification of studies through keyword-based queries, removal of duplicates, screening stage based on title and abstract, eligibility assessment through full-text analysis, and final inclusion of studies deemed relevant to the field.

Additionally, an accessibility filter was applied by selecting only open-access publications to ensure a detailed analysis of articles. This resulted in a final set of 407 articles for WoS, 429 for Scopus, and 34 for IEEE Xplore.

For the set of criteria for classifying ML algorithms, we used a mutually exclusive classification scheme, where each algorithm is assigned to a single main category based on its fundamental nature, rather than its application domain. In this sense, the algorithms were divided into the following main categories: supervised learning, unsupervised learning, and deep learning. DL models (e.g., Convolutional Neural Networks—CNN and Recurrent Neural Networks—RNN, including Long Short-Term Memory—LSTM) have been treated as a distinct category and have not been included simultaneously in other categories, even if they are used for specific tasks such as time series prediction. This approach is justified by the attempt to avoid overlaps. Thus, the classification was not based on the type of problem (e.g., regression, classification, and time series analysis), but exclusively on the algorithmic architecture. Thus, models like LSTM were strictly categorized as deep learning, even though they are frequently used for time series modeling. This approach ensures the separation between categories and eliminates ambiguities resulting from multiple classifications of the same algorithm.

The selection of open-access publications (407 WoS articles, 429 Scopus, and 34 IEEE Xplore) introduces a selection bias. In the context of this analysis, the bias could affect the distribution of algorithms (e.g., the overrepresentation of popular methods in open science communities) or tasks (e.g., an emphasis on prediction over calibration), but the research trend of ML in relation to IAQ remains the same. The reason for focusing the analysis exclusively on WoS and open-access articles comes from the content analysis of the articles. The authors of the present article aimed to capture how researchers relate, firstly, to the issue of IAQ and, secondly, to a specific database.

The included studies were not formally weighted based on methodological quality or the validation strategy used. The objective of the analysis was to identify methodological trends and the distribution of algorithms in the field. For these reasons, the research does not conduct a quantitative meta-analysis. However, in interpreting the results, the size of the datasets, the type of validation (cross-validation, hold-out, and inter-seasonal test), the transparency of reporting indicators, and future trends were taken into account.

## 4. ML Survey Through the WoS Literature on IAQ

In indoor air quality research, modeling tasks tend to fall into several recurring categories: (i) exposure/health association and risk-factor analysis, (ii) sensor calibration and proxy estimation (e.g., estimating PM_2.5_ from low-cost sensors and CO_2_ as ventilation proxy), (iii) forecasting (short-term prediction of pollutant levels and IAQI), and (iv) source or feature extraction and dimensionality reduction. The algorithms with high WoS counts are generally those that map well onto these tasks while also being easy to justify to multidisciplinary audiences (engineering with public health and building science), and easy to implement with typical IAQ datasets (moderate sample sizes, multicollinearity, missingness, strong seasonality, and mixed physical or behavioral drivers).

**Figure 3:**
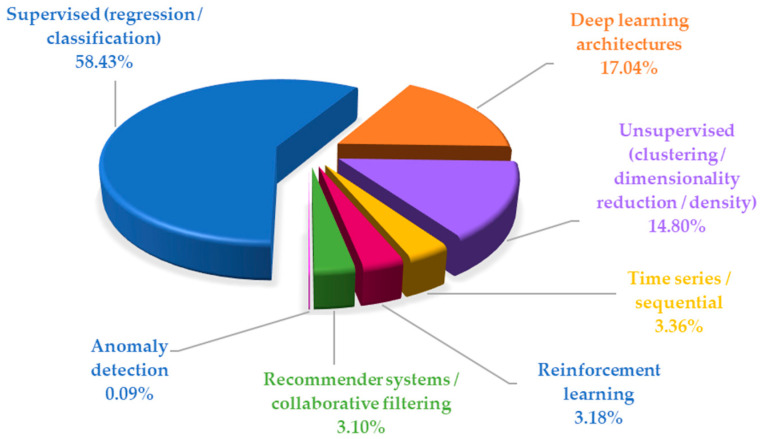
Weight of the ML algorithms categories among published papers (2020–2025).

The queries on the WoS database generated 1162 papers, and more than half (58.43%) have implemented ML algorithms from the supervised (regression/classification) category, followed by deep learning architectures (17.04%), and unsupervised (clustering/dimensionality reduction/density) (14.8%). Similar weights are among time series/sequential (3.36%), reinforcement learning (3.18%), and recommender systems/collaborative filtering (3.10%). Anomaly detection was used in the lowest proportion of 0.09%. The categories in Figure 3 are mutually exclusive, based on the algorithm’s architecture, not on the type of task. DL is treated as a distinct category to avoid double-counting of neural networks with multiple layers. Mapping algorithms in Figure 3 contain supervised models (Ordinary Least Squares—OLS, Logistic Regression—LR, RF, Elastic Net, SVM, k-Nearest Neighbor—kNN, DT—Decision Tree, eXtreme Gradient Boosting—XGBoost, etc.), DL models (LSTM, CNN, Multilayer Perceptron—MLP, RNN, Gated Recurrent Unit —GRU, etc.), unsupervised models (Principal Component Analysis—PCA, k-means, etc.), and time series models (Seasonal Autoregressive Integrated Moving Average—SARIMA, Error Trend Seasonal—ETS, etc.). The entire list of the investigated models is presented in the Supplementary Materials section (Investigated_models.xlsx).

**Figure 4:**
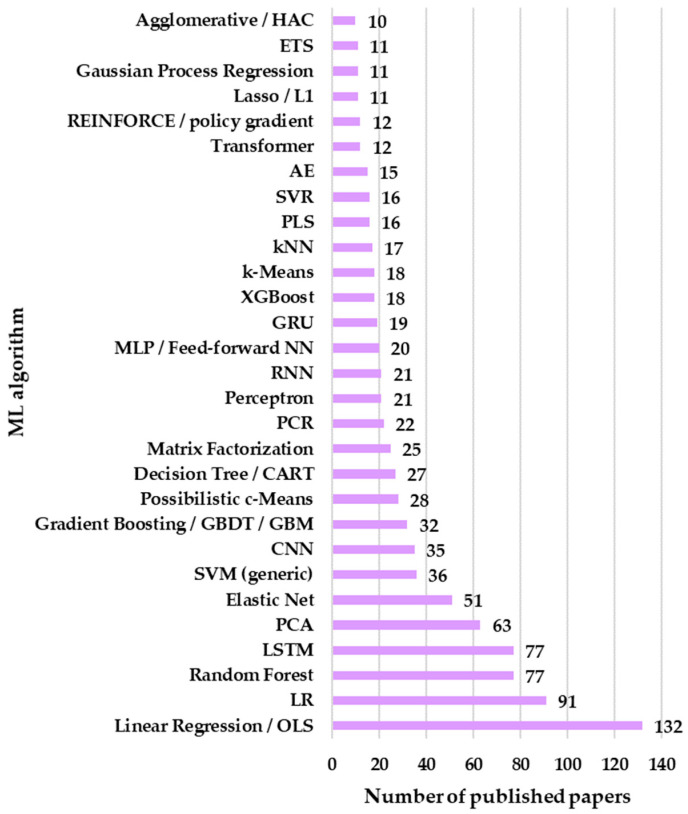
Most implemented ML algorithms in published papers (2020–2025). Note: AE—Autoencoder; CART—Classification and Regression Tree; GBDT—Gradient Boosting Decision Tree; GBM—Gradient Boosting Machine; HAC—Hierarchical Clustering; PCR—Principal Components Regression; PLS—Partial Least Squares; SVR—Support Vector Regression.

Of the 175 ML algorithms, only 100 are used in indoor air quality research papers, and those implemented in more than 10 studies are highlighted in Figure 4. In cases where papers employed multiple algorithms, each algorithm was counted and reported separately to ensure an accurate picture of the results.

The dominance of OLS, also known as linear regression, generated 132 results. These values indicate that a large share of IAQ studies still prioritize interpretability, effect estimation, and hypothesis testing over pure predictive accuracy. This aligns strongly with typical IAQ questions, such as:

- Quantifying the impact of ventilation rate, occupancy, temperature/RH, outdoor infiltration, and activities on indoor pollutant concentrations;
- Estimating exposure–response relationships and reporting coefficients in physically interpretable units;
- Building baseline models for calibration or benchmarking.

The relatively high presence of Elastic Net (51 articles) is also revealing. IAQ datasets often include correlated predictors (e.g., temperature, RH, heating, ventilation, and air conditioning (HVAC) state, occupancy proxies, outdoor PM, and window opening), and Elastic Net directly addresses multicollinearity while performing embedded feature selection. Its prominence suggests the community is frequently dealing with “wide” covariate sets (sensor arrays, building metadata, contextual weather variables, etc.) where a plain OLS specification becomes unstable or difficult to interpret.

The strong count for LR (91 articles) points to the popularity of classification framing in IAQ for predicting whether a space is in a “good vs. poor IAQ” state, whether pollutant thresholds are exceeded, whether ventilation is adequate, or whether an intervention is needed. This also corresponds to a practical reality because many IAQ outputs are naturally discretized into categories (e.g., acceptable/unacceptable, risk/no-risk, comfort complaint yes/no, etc.) or threshold-based events (e.g., PM_2.5_ > guideline). LR remains attractive because it offers interpretable odds ratios, straightforward handling of mixed categorical/continuous inputs, and regulatory or guideline-aligned “decision” outputs.

The appearance of RF (77 articles) and GB (32 articles) indicates that many IAQ studies have moved beyond linearity to capture nonlinear relationships (e.g., RH effects on particle sensors, ventilation dynamics, occasional events like cooking, etc.), interactions (occupancy and ventilation, outdoor pollution and infiltration, and HVAC mode and season) and robustness to missingness/noisy features common in Internet of Things (IoT) deployments. RF’s higher count relative to generic GB likely reflects ease-of-use and “default” adoption. RF is frequently used as a strong reference with minimal tuning, and it handles mixed feature types well. GB models are also common but are sometimes reported using library-specific names (e.g., XGBoost, LightGBM, CatBoost, etc.), which can fragment counts across queries—meaning the 32 for generic boosting likely underestimates boosting’s total footprint.

That LSTM equals RF (77 articles) is a strong indicator that sequence modeling/forecasting is an important IAQ trend in 2020–2025. IAQ time series are characterized by strong temporal autocorrelation and daily/weekly cycles, abrupt activity-driven spikes (cooking, cleaning, occupancy changes, etc.), and lagged relationships (CO_2_ accumulation, delayed decay, HVAC response, etc.). LSTMs are commonly selected because they explicitly model temporal dependencies and can be presented as a “forecasting engine” for short-term prediction of PM_2.5_, CO_2_, VOC proxies, or IAQI.

The presence of CNN (35 articles) suggests a second deep learning pathway by using convolutions to learn patterns in either (i) time series windows (one-dimensional Convolutional Neural Network—1D CNN) or (ii) transformed representations (spectrogram-like encodings, multivariate matrices, etc.). CNNs often serve as efficient feature extractors for high-frequency sensor streams and components in hybrid CNN-LSTM pipelines used for forecasting and event detection.

LSTM/CNN counts show that the field increasingly treats IAQ as a dynamic signal-processing problem (forecasting, early warning, event detection, etc.) rather than only a static regression problem. However, the fact that these deep models do not overwhelmingly exceed classical methods also suggests practical constraints, for instance, limited labeled data, site-specific generalization issues, and the ongoing need for interpretability in building operation contexts.

The high count for PCA (63 articles) reflects two persistent IAQ needs handling multivariate sensor sets (multiple pollutants and environmental variables) by compressing information into a few latent components and source/process interpretation, where principal components are used as proxies for shared drivers (e.g., “occupancy/ventilation component”, “outdoor infiltration component”, “combustion event component”, etc.). PCA is also commonly used in preprocessing for subsequent regression or classification, for denoising, and for visualization/cluster exploration. Its prominence indicates that many IAQ datasets are inherently multicollinear and multi-sensor, and that researchers value low-dimensional representations that can be inspected and explained.

The SVM (36 articles) count suggests that SVMs continue to be used for IAQ classification/regression problems where datasets are not huge (SVMs remain competitive in small-to-medium sample settings), decision boundaries are expected to be nonlinear (kernel SVM), and there is emphasis on robust separation between “acceptable vs. unacceptable” conditions.

It must be noted that SVM (generic) appears while SVC and some specialized variants show zero in the query set. This is because authors often write “SVM” without specifying the exact scikit-learn class name or formal variant.

Taken together, the algorithms exceeding 30 records form three complementary methodological “centers of gravity” in IAQ:

1. Inference-first, interpretable modeling (OLS, Logistic, Elastic Net, etc.) indicates continued emphasis on understanding drivers of indoor pollution and producing results that can be communicated as effects, risks, and actionable factors;
2. Robust nonlinear prediction on heterogeneous data (RF, GB, SVM, etc.) replicates the practical reality of IoT-based IAQ (noisy sensors, missing data, nonlinearity, and site-specific interactions);
3. Time-aware deep learning (LSTM and CNN) reflects growth in forecasting, early warning, and control-oriented applications (predictive ventilation and smart building operation), where temporal structure is central.

PCA sits somewhat orthogonally but remains highly prevalent because it supports both interpretability (latent drivers) and predictive pipelines (compression/denoising).

Finally, these WoS counts should not be read as a ranking of the best algorithms for IAQ. They are more accurately interpreted as a combined measure of:

- Method popularity and accessibility;
- Fit to common IAQ problem formulations (threshold classification, multivariate regression, short-term forecasting, etc.);
- Disciplinary conventions (public health and building science favor interpretable regressions, etc.);
- Keyword and reporting practices, because some methods appear under many names, whereas others are used but not explicitly labeled.

To ensure objective benchmarking and reproducibility, we complement the IAQ measurements with openly publicly available datasets that provide multi-sensor time series across diverse buildings, climates, and occupancy patterns, enabling model training and fair comparisons.

Classification and regression are two components of ML. Classification categorizes the value of the output variable into a category from a predefined set of categories. Classification performance is measured by accuracy. This is defined as the percentage of correct predictions [16].

Regression, on the other hand, predicts continuous values, and its quality is evaluated with R^2^. This metric represents a coefficient of determination that evaluates how much of the data variation is explained by the model. R^2^ = 1 means a perfect prediction. An R^2^ value of 0 or a negative value indicates a model that does not perform well in applications.

Table 2 shows the accuracy (from 85–yellow to 99.37–dark green) reported in several scientific papers, correlated with the research objective and the ML algorithm type employed. When a paper reported multiple accuracy values, the highest value was chosen. Most studies evaluating IAQ using classification or regression report accuracies over 90%, with peaks of 99.37% (IAQ classification with LSTM [17]) and 99.20% (ventilation prediction with RF [18]). Hybrid models (LSTM/SARIMA—95% [19]) or meta-learning-based models (94.93% for PM_10_ [20]) highlight the possibility of combining models to improve performance. Classic ensemble algorithms (RF and XGBoost) generate good performance in most domains, a fact that is also confirmed in the case of IAQ (91–95% [21,22]). The lowest values appear in complex contexts: radon (85% [23]), due to spatial heterogeneity, or bioaerosols (90% [24]).

Out of the total evaluated articles, the studies that reported explicit numerical accuracy, an R^2^ of over 70%, were retained. The authors imposed this threshold value because it is associated with a minimum level of predictive performance. Values below this level are considered models with predictive deficiencies, and it is not recommended to integrate them into real applications for monitoring indoor air quality.

Studies show that regression models in IAQ parameter prediction have good computational capacity for the target variable value, as demonstrated by R^2^. As can be seen in Table 3, the prediction of CO_2_, PM_2.5_, or other IAQ parameters reports R^2^ values between 72.2–dark blue and 98.9–dark red. When a paper reported multiple R^2^ values, the highest value was chosen. The best performance (98.90%) comes from a hybrid model based on XGBoost [28]. LSTM or RF-LSTM architectures achieve over 98% accuracy in CO_2_ prediction [29]. RF-based models stand out for their R^2^ values, which range between 83% and 95%. Lower values (72–73%) appear in contexts with noisy data. An example of this is the estimation of aerosol emissions [30,31].

In classification, besides accuracy, other performance metrics such as precision, recall, and F1-score are also used. Precision shows the proportion of correct positive predictions, recall measures how well all positive instances are identified, and the F1-score is their harmonic mean.

Other metrics for regression are mean absolute error (MAE) and root mean squared error (RMSE). MAE measures the average error in the same unit as the target. RMSE penalizes errors. The choice of the appropriate metric depends on the context: in IAQ applications, for example, RMSE is the benchmark if large errors have an impact on health [44]. On the other hand, the F1-score is mandatory in situations where the rare detection of critical pollution states is required. Mean absolute percentage error (MAPE) is a metric used in regression to evaluate the accuracy of predictions expressed as a percentage. Overall, the results confirm that under controlled conditions, ML classification and regression provide predictions for IAQ that can be used in practice.

## 5. IAQ Public Datasets

This section reviews representative public IAQ datasets, outlining their sensor modalities (e.g., CO_2_, PM_2.5_, VOCs, temperature, RH, etc.), sampling rates, and labeling strategies, and discusses how they can be harmonized into ML tasks.

*1. Indoor Air Quality Dataset with Activities of Daily Living in Low to Middle-income Communities* (DALTON dataset) is a public IAQ dataset [45,46]. It contains a large-scale IAQ dataset covering 30 unique indoor sites across low to middle-income communities in India with annotated human activity [47]. The parameters included are pollutants (CO_2_, VOC, PM_1_, PM_2.5_, PM_10_, NO_2_, C_2_H_5_OH—ethanol, and CO), environmental measurements (temperature and RH), and activity annotations (daily activities collected via speech-to-text). The dataset size contains approximately 89.1 million samples, 13,646 h of recorded data, and 3900 activity annotations.

Multi-site real sensor data from multiple indoor environments corresponds to households, classrooms, labs, food canteens, and annotated human behavior tags. Across the IAQ experiments, the strongest results are achieved by tree-based methods and kNN, with very high weighted F1-score, precision, recall, and strong cross-validation stability. The weaker family is Naive Bayes, the linear models, and the SVMs sit in the middle.

The DT corresponds to the top scores with the following results: (i) train (weighted), where F1-score, precision, and recall have the values at 99.2%; (ii) test (weighted), where F1-score, precision, and recall obtained the values of 97.6%. This is the best-performing model family mentioned in a paper [48].

Similar results are obtained for RF, with best test performance reported at 98.8% for F1-score, precision, and recall in the training stage and 97.7% for F1-score, precision, and recall in the testing stage [48]. RF is tied with the best models on the test set. It also typically generalizes more than a single DT, even if here the single tree is marginally higher.

The kNN (k = 10) algorithm is near the top and very consistent with train metrics at 98.1% and test metrics at 97.5%. The k = 10 provides the best kNN configuration. As k increases (20 to 40), performance gradually drops, suggesting that too much smoothing hurts class separation.

*2. Indoor Air Pollutants Using Low-Cost Sensors* [49] is a public dataset. The low-cost IoT system [50] is an extended version of the first. The datasets represent indoor pollutant measurements collected over multiple seasons in Pune, India, using low-cost air quality IoT sensors.

Parameters included for the first dataset are pollutants (PM_2.5_, NO_2_, NH_3_, CO, and O_3_) and environmental measurements (temperature, RH, and pressure). The dataset contains approximately 173,468 records from November 2020 to July 2022. The dataset was analyzed by Singh et al. [51], where it was reported as having missing pollutants with high impact on IAQ. The authors also reported missing values with high impact on ML models.

However, Ali et al. [52] analyzed the [49] dataset. The main contribution is combining sequence models (a Hybrid LSTM-GRU) with Liquid Neural Networks (LiquidAdaptedNet) and then optimizing and refining the liquid architecture using swarm intelligence and evolutionary refinement. The authors [52] report that the evolutionary-refined (swarm-optimized) LiquidAdaptedNet achieves the best headline regression metrics, RMSE of 6.52, MAE of 6.27, R^2^ of 97.63%, and MAPE of 1.41. These values indicate high predictive accuracy. A major practical result is that the proposed framework provides accurate forecasts for three key indoor pollutants: NO_2_ up to 99.97% (forecast performance, depending on operating conditions), PM_2.5_ up to 98.67%, and CO up to 94.47%. Performance is extremely strong for NO_2_ and PM_2.5_, and still high for CO (but comparatively lower), suggesting CO dynamics might be harder to capture (e.g., different emission patterns, sensor noise, or more abrupt changes, etc.). The dataset [50] was also analyzed by Sonawani and Patil [53]. The conclusion of the research mentions a 55.42% RMSE score for prediction.

*3. IoT Indoor Air Quality Dataset* [54] is mentioned as a real-time IAQ dataset collected with multiple IoT sensors under controlled indoor conditions. Parameters included are pollutants (PM_2.5_, PM_10_, CO_2_, TVOC, and CO) and environmental measurements (temperature, RH, light intensity, motion detection, the number of occupants, and ventilation status).

According to the authors’ statements, the dataset contains real-time data collected from an IoT sensor network deployed in a residential environment, specifically inside an apartment, and the data was collected between 18 February 2024 and 22 January 2025, totaling 97,458 records. This is a temporal analysis of IAQ at the residential level. According to the authors’ statement, the dataset was constructed using sensors installed at a height of approximately 1.5 m to simulate the position of the human respiratory zone. To minimize external interference, the sensors were placed far from open windows, exhaust fans, air conditioning units, or ventilation elements. The dataset includes variations in air quality associated with household activities such as cooking, vacuuming, etc., changes in occupancy levels, ventilation status, or other parameters specific to indoor activities that cause changes in temperature, RH, and light levels [54]. However, the dataset is mentioned in the literature only once [55], and it was not included in ML tests.

*4. GAMS Indoor Air Quality Dataset* [56] is publicly available and contains synchronized indoor and outdoor air quality measurements collected by the GAMS monitoring ecosystem. Indoor data is recorded using a GAMS Indoor Air Quality Monitor, while outdoor air quality data is retrieved via the GAMS Outdoor Air Quality API. The dataset is designed for real-time and historical assessment of indoor–outdoor pollution relationships. Parameters included are pollutants (PM_2.5_, PM_10_, CO_2_, and VOC) and environmental measurements (temperature and RH).

The dataset is time-series-based and typically grows continuously depending on monitoring duration and sampling frequency. The dataset contains, on 7 January 2026, a total of 135,100 records. The dataset was identified as being analyzed in the papers [57,58]. This study [57] investigates how ML and DL methods predict indoor CO_2_ concentration. Using the GAMS dataset, the authors modeled CO_2_ levels. The inputs are PM_10_, PM_2.5_, temperature, RH, and VOCs. All models were evaluated under the same experimental protocol, 5-fold cross-validation with an 80% training and 20% testing split. A broad set of predictive approaches was tested. Traditional ML models included Decision Tree Regression (DTR), RF, kNN, SVR, and Gradient Boosting Regression (GBR). At the same time, several DL architectures were examined, such as LSTM, CNN, GRU, Bi-LSTM, hybrid variants like RF-LSTM and CNN-LSTM, and an additional DL approach referred to as DAQFF. This diverse benchmarking framework allowed this study to assess both conventional tabular-learning techniques and sequence-focused neural models suited for time series behavior. The comparative results highlight GBR as the best performer for predicting indoor CO_2_ concentration. GBR achieved the following error values: MAE of 0.00200, RMSE of 0.00353, Median Absolute Error of 0.00086, and an R^2^ value close to 1, indicating a near-perfect fit on the evaluated data. Among deep learning methods, LSTM also demonstrated high performance, reaching an MAE of 0.001661, which reinforces its well-known strength for time series prediction where temporal dependencies matter. Overall, the findings highlight that both boosting-based ML and recurrent DL models can provide highly accurate CO_2_ forecasts, offering practical value for monitoring and managing indoor environments, particularly in contexts associated with “sick building” concerns.

*5. This dataset* [59] provides aggregated daily indoor and outdoor environmental variables measured at three school sites in Milan (Italy) between November 2023 and June 2024, in settings where professional air purifiers were installed in some monitored indoor environments. Indoor measurements come from multiple low-cost sensors and were aggregated to a single daily value per site. Outdoor pollution indicators were derived from the European Environment Agency (EEA) monitoring network by selecting the two closest background stations to each school and applying inverse distance weighting (IDW) to obtain representative daily values.

The dataset contains 627 records, which is not suitable for ML techniques. The parameters are pollutants (PM_2.5_, PM_10_, CO, and CO_2_), outdoor pollutants EEA-derived and IDW from nearest stations (PM_2.5_ and O_3_), and environmental measurements (temperature, RH, and indoor atmospheric pressure). There are no scientific papers in the ML area.

*6. The dataset available at source* [60] and described by [61] contains multi-sensor, time-resolved indoor environmental measurements collected in classrooms from schools and universities over several months. It is designed to support research on indoor air dynamics, ventilation effectiveness, and the impact of sensor placement on measurement reliability (especially for CO_2_). The dataset contains 11,086 records. Parameters include pollutants (PM_1_, PM_2.5_, PM_10_, CO_2_, and VOC) and environmental measurements (air temperature, RH, dew point, globe temperature, air pressure, air velocity, occupancy, activity, and ventilation). The dataset is not involved in ML tests on 7 January 2026.

For public datasets, information on the proportion of missing values and any sensor calibration methods was included where available. Additionally, the sources of access to the datasets were indicated to facilitate their use by other researchers. In situations where this information was not explicitly reported in the analyzed papers, the corresponding fields were marked as “Not reported” to avoid introducing unjustified assumptions.

Table 4 presents the synthesis of the analyzed datasets, summarizing the parameters and dataset volumes.

The analysis of structural characteristics delineates the following use cases:

- DALTON is used for studies of the association between human activities and pollutant dynamics, due to its large volume (~89 M records), generous number of features, diversity of locations, and verbal labels (speech-to-text). The authors of this article do not recommend using this dataset for analysis that requires internal–external synchronization.
- GAMS analyzes the correspondence between indoor and outdoor pollution for the validation of infiltration or ventilation models. This dataset stands out from the others because it is the only one that synchronizes indoor–outdoor measurements through a dedicated API.
- Low-cost IoT is used for seasonal studies, calibration of low-cost sensors, evaluation of imputation algorithms, and multi-year coverage. A drawback of this dataset concerns the need for preprocessing missing values.
- IoT indoor air quality is suitable for short-term modeling of discrete events (cooking, ventilation, and occupant variations) in controlled environments, with high temporal resolution but limited collection duration.
- School IAQ Milan and Multi-Sensor Classroom evaluate the ventilation capacity within classrooms, the impact of air purifiers, multiparameter analysis, and sensor positioning (especially CO_2_). A major disadvantage of this dataset arises from its small size, making it inadequate for ML/DL architecture and more suitable for linear models or conceptual validations.

The choice of the dataset must reflect the temporal granularity, the availability of contextual variables through occupancy, ventilation, external references, and the presence of labels. The improper use of these resources generates generalization errors. Another consequence is the limitation of model transferability between different buildings.

Overall, public IAQ datasets offer a strong foundation for validation and transfer learning, but differences in sensors, calibration, metadata quality, and ground-truth definitions require careful preprocessing and transparent reporting to ensure meaningful cross-study comparisons and reliable conclusions [62].

## 6. IAQ Private Datasets

Most IAQ research between 2020 and 2025 relies on custom, sensor-based experimental setups rather than a single standardized monitoring configuration. Authors typically design their own multi-sensor nodes by combining low-cost sensors with a small number of higher-accuracy references. This is reflected by the diversity of sensing principles used for different pollutants and by the uneven publication counts across parameters. Researchers focus strongly on “core IAQ” variables (PM, VOCs, CO_2_, temperature, and RH) and far less on auxiliary context variables (pressure and illuminance) or on specific gases with more challenging sensing (NH_3_, C_2_H_5_OH, and NO_2_).

Next is an overview of which sensors are commonly used for each parameter, and a commentary on the search query result counts (TS… AND PY = 2020–2025). CO_2_ uses the Non-Dispersive InfraRed (NDIR) sensor typically. CO_2_ absorbs infrared radiation at specific wavelengths, allowing selective measurement with good stability and relatively low cross-sensitivity compared to many chemiresistive approaches. The query TS = ((“indoor air quality” OR “iaq” OR “iaqi”) AND (“CO2” OR “Carbon dioxide”) AND (“NDIR” OR “Non-Dispersive InfraRed” OR “CO2 sensor*”)) AND PY = (2020–2025) generates 71 articles. CO_2_ is widely monitored indoors because it is a strong indicator of ventilation adequacy and occupancy-related emissions. However, CO_2_ monitoring is often “embedded” in broader IAQ studies that may not explicitly highlight NDIR in titles, abstracts, or keywords.

VOC or total volatile organic compound (TVOC) uses metal oxide semiconductor (MOS) chemiresistive gas sensors, usually reported as VOC or TVOC rather than individual VOC species. Metal-oxide (MO_x_) sensors are low-cost, compact, with reduced dimensions, and easy to integrate into IoT nodes. They respond to many oxidizing gases. This relatively high count (33 articles) matches the popularity of VOC sensing in IAQ. Many studies use TVOC as a general proxy for indoor chemical pollution. The literature often addresses drift, cross-sensitivity to RH or temperature, and calibration strategies (e.g., ML compensation). The count is still below PM studies because VOC sensing is messier, that is, the signal is less interpretable and more dependent on the environment and mixture composition.

Particulate matter (PM_1_/PM_2.5_/PM_10_) is measured using optical particle counters (OPC) based on laser or light scattering. Optical scattering sensors provide real-time particle concentration estimates at low cost and are widely deployed in indoor monitoring and smart building applications. A total of 52 papers mentioned the use of such a sensor. This value is consistent with the strong research focus on PM_2.5_ and health impacts.

NO_2_ is measured using electrochemical or MO_x_ sensors. Electrochemical sensors are generally used for NO_2_ measurements. MO_x_ sensors respond to NO_2_ but are often more susceptible to interference from other oxidizing or reducing species and to environmental influences such as temperature and RH. The relatively small corpus (9 articles) indicates that NO_2_ is less commonly emphasized in “general” IAQ research. This may reflect the fact that indoor NO_2_ levels are often highly context-dependent (associated, for example, with combustion sources such as gas cookers or with infiltration from nearby traffic) rather than being a ubiquitous IAQ parameter across building types. In addition, achieving robust low-cost NO_2_ measurements remains technically challenging due to calibration requirements, cross-sensitivities, baseline drift, and long-term stability concerns. Consequently, many studies may address NO_2_ primarily within the framework of outdoor air pollution ingress and exposure, rather than as a central variable in mainstream IAQ sensing.

C_2_H_5_OH uses MO_x_ chemiresistive sensors (often the same class used for VOC detection). C_2_H_5_OH is a calibration or test gas for MO_x_ sensors and an indoor compound (cleaning agents and disinfectants). Very low results (3 articles) are expected because C_2_H_5_OH is rarely monitored as a standalone IAQ target. It is more often discussed as a contributor to TVOC or as an interferent or benchmark gas during sensor characterization.

CO involves electrochemical amperometric CO sensors. Many IAQ studies include CO only incidentally, focusing instead on CO_2_, PM, VOC, and thermal comfort. The query’s explicit (3 articles) emphasis on electrochemical terms may also filter out papers that do not specify the sensor type.

Temperature uses thermistors, resistance temperature detectors (RTDs), or integrated micro-electro-mechanical systems (MEMS) temperature sensors (often on combined T/RH chips). Temperature is easy to measure accurately with low-cost electronics, and it helps interpret other sensors (VOC/PM responses) for comfort metrics. High counts (27 articles) make sense because temperature is nearly always included as a contextual variable in IAQ nodes and is frequently mentioned explicitly. Temperature is also a key variable for compensating gas-sensor drift and RH interactions.

Humidity or RH (capacitive/resistive/MEMS sensors) generates 16 results. Capacitive humidity sensing is stable, low-power, low-cost, and widely available in integrated modules. Humidity appears frequently, but possibly less than temperature, because many papers treat RH as a secondary parameter unless they focus on mold risk, perceived comfort, or sensor compensation. Also, RH measurement is often bundled with temperature on a single chip, which may reduce the need for explicit keyword emphasis.

NH_3_ was studied in only 7 records. The monitored IAQ parameter is NH_3_ concentration, measured using electrochemical NH_3_ sensors and MO_x_ or MOS chemiresistive gas sensors (ammonia sensors).

Pressure, or barometric pressure or air pressure, is measured with a barometric sensor or MEMS pressure sensor (barometer). However, no records were found for this query.

The light intensity is measured with a photodiode-based light sensor, photoresistor, or ambient light sensor. Again, no records were found for this query.

The monitored IAQ-relevant parameter associated with the occupant presence or motion (as a proxy for ventilation demand and pollutant generation) is measured primarily using passive infrared (PIR) sensors. Only two articles were identified in the WoS query search.

The occupancy level (number of people in the room) is measured using computer-vision approaches (camera and deep learning techniques) and PIR sensors, ultrasonic sensors, or sensor-fusion combinations integrating multiple modalities for occupant counting.

These results show the preference for certain parameters, such as PM, VOC/TVOC, and thermal variables, followed by CO_2_ and occupancy-related methods. Some of the parameters (NO_2_, NH_3_, C_2_H_5_OH, and CO) indirectly contribute to the IAQ calculation. The specialized literature provides a relatively limited detailed description of the infrastructure used in air quality studies.

## 7. IAQ–ML Approach with Sensors

**Figure 5:**
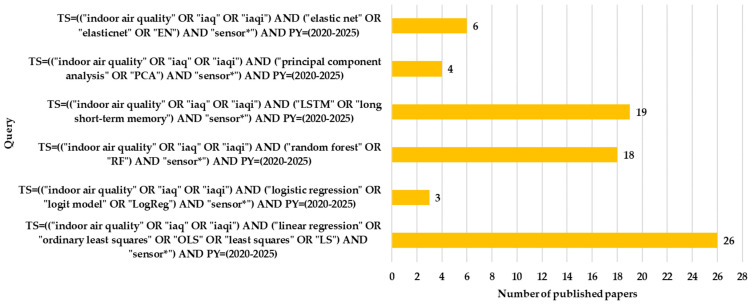
Results on investigating IAQ using sensors for data acquisition between 2020 and 2025 (the asterisk in the word “sensor” was used to include all lexical derivatives).

Search results investigating IAQ using custom-made solutions that include sensors for data acquisition are discussed. The methodological trends in sensor-based IAQ research between 2020 and 2025 are illustrated in Figure 5. Linear regression remains at the top of the most used ML option (26 studies). In contrast, LR is surprisingly rarely used (only 3 studies). This result is justified by the fact that IAQ often involves continuous predictions (e.g., concentrations), not binary classifications. Modern models dominate through RF (18 studies) and LSTM (19 studies). The low value presence of PCA (4 studies) suggests that dimensionality reduction is used selectively in preprocessing. Elastic Net appears in 6 studies. As a consequence, there is a shift from simple linear models toward complex architectures (RF and LSTM), without completely abandoning classical approaches. This methodological diversity highlights the maturity of the IAQ field in integrating sensors with ML.

Across 2020–2025, the retrieved IAQ literature is dominated by IoT sensing and ML, most frequently LSTM variants for short-horizon forecasting, plus tree ensembles (RF/XGBoost) for prediction under event-driven dynamics, and PCA for sensor or feature validation. The studies differ in the target variable because some forecast pollutant concentrations (CO_2_, PM_2.5_, and VOCs), others predict an index (IAQI) or even occupant dissatisfaction (Percentage Dissatisfied—PD%), and several focus on sensor calibration or anomaly detection.

A common thread is the creation of low-cost monitoring systems and the attempt to replace expensive reference instruments with either improved hardware plus ML, or virtual sensing from building management system (BMS) data. For example, an IoT CO_2_ monitoring platform uses Message Queuing Telemetry Transport (MQTT) streaming and an LSTM to predict future CO_2_, enabling proactive ventilation. It reports the CO_2_ steady-state computed in advance with ~5.5% error [63]. Similarly, a classroom-oriented low-cost NDIR system optimized an LSTM architecture (two layers, 128 neurons, 10-lag window) and achieved RMSE ≈ 57 ppm for an 8-h CO_2_ forecast [64]. Several works extend beyond single-room demonstrations into long-term deployments, capturing seasonality. In a hospital setting with nearly one year of IoT data, clustering outputs were fed into an LSTM to improve prediction. The key numerical result is a large reduction in PM_2.5_ RMSE from 8.51 to 3.99 when cluster labels or features were included [65]. This suggests that unsupervised structure helps the temporal model separate dynamics that would otherwise appear noisy. A similar hybridization idea appears in smart-village monitoring, where k-means is used to cluster IAQ states, and a Markov-based model predicts state transitions. The optimal number of Markov states differed by building type (e.g., 3 states for schools versus 5 for supermarkets), and the paper reports example state values such as polluting gas 862.6 ppm, temperature 28.66 °C, and light 70.05 lux for a supermarket condition [66].

Another direction is virtual sensing, where models infer pollutant levels without a dedicated pollutant sensor. An LSTM trained on multi-year data predicted indoor CO_2_, PM_2.5_, and VOC from BMS variables plus outdoor meteorology or pollution, achieving in-room test performance of MAE 15.4 ppm (CO_2_), 0.3 µg/m^3^ (PM_2.5_), 20.1 IAQI (VOC), and R^2^ = 0.47 (CO_2_), 0.88 (PM_2.5_), 0.87 (VOC). Transfer to other rooms remained acceptable for CO_2_ (MAE of 21.9 ppm and R^2^ of 0.45) but degraded sharply for PM_2.5_ and VOC (R^2^ of 0.09 and 0.13) [67]. Some studies prioritize sensor calibration rather than direct IAQ forecasting. In Korean daycare centers, an LSTM-based calibration model for low-cost PM_2.5_ sensors (using temperature–humidity as predictors) reached R^2^ of 0.962, MAE of 2.7, and reported error terms, including RMSE of 3.57 (and a mean squared error—MSE—reported as 12.745) at an optimal lookback of 76 [33]. Importantly, it substantially outperformed linear baselines (R^2^ of 0.57 for linear regression and 0.75 for multiple linear regression) [33], supporting the notion that low-cost optical PM sensors’ nonlinear dependence on RH and other factors can be learned effectively by sequence models. In residential monitoring, the key empirical insight is that short peaks matter even if 24-h means look safe. A dataset from three domestic micro-environments (kitchen/living room/bedroom) captured peaks such as bedroom CO_2_ > 2800 ppm and PM spikes during cooking [68]. For occupant-centric outcomes, one school IAQ study predicts perceived PD% from sensor data plus weather or building parameters and 1437 surveys. RF was best, reaching R^2^ up to 0.91 for overall dissatisfaction and outperforming multilinear regression and DTs [38]. SHapley Additive exPlanations (SHAP) prioritized CO_2_ levels, VOCs, RH, temperature, solar radiation, and window orientation [38], quantitatively backing the multi-factor nature of perceived comfort. In a different cross-zone apartment problem, kitchen sensors were used to predict living-room conditions via multiple-input multiple-output models. Surprisingly, linear methods performed best, achieving R^2^ of 0.94 (temperature), 0.94 (RH), 0.63 (CO_2_), 0.84–0.92 (TVOC), with low errors for temperature (MAPE of 1.57%) and RH (MAPE of 2.97%) but high error for CO_2_ (MAPE of 20.83%) [35]. This numeric pattern is plausible because temperatures are more spatially coupled across zones, while CO_2_ depends on localized occupancy and air exchange, making it harder to infer from a different room.

A few works emphasize chemical specificity and feature engineering rather than classical IAQ targets. Using low-cost VOC sensors plus ML, one office testbed trained RF/SVM/XGBoost to detect terpene presence and identity. The performance was very high, with 97–100% accuracy for any terpene versus background, ~100% for plants vs. background, and up to 96% for discriminating individual terpene compounds [25]. Living-plant experiments observed stress-induced bursts of ~70–100 ppb [25], illustrating that ML extracts compound-aware signals from commodity TVOC hardware when paired with time series features.

Traffic-related air pollution in a Riyadh school was studied using PCA and correlation analysis. Indoor PM_2.5_/PM_10_ averages were 14.09 and 18.15 µg/m^3^ vs. outdoor 20.63 and 27.88 µg/m^3^, with strong indoor–outdoor coupling (Spearman 0.81–0.94). Indoor NO_2_ was higher (43.36 ppb) than outdoor (38.66 ppb) [69], indicating indoor sources or ventilation inadequacy. PCA ranked PM as the dominant contributor [69]. Likewise, an electro-optical e-nose used PCA for separability and PLS regression for concentration prediction, and by adding NDIR to MO_x_ sensors, reduced RMSE dramatically [70]. The paper by González et al. [70] is a quantitative argument for multi-sensor fusion in low-cost IAQ instrumentation.

Several contributions broaden the systems perspective. An indoor IAQI forecasting system in Mexico City compared 133 deep models and found LSTM best, achieving MSE of 0.0179 and MAE of 0.1038 for IAQI forecasting using indoor sensor and outdoor pollution and weather, with approximately 5 months of history sufficient and relatively small networks (~50k parameters) adequate [71]. A smart-building platform combined SARIMA and LSTM for energy forecasting (and IAQ control context), reporting MSE of ~0.01 for energy predictions [72], reinforcing the coupling between IAQ control strategies and energy management.

Across the 2020–2025 IAQ sensor literature, the dominant uses of data-driven models are (i) forecasting IAQ variables, (ii) calibrating low-cost sensors vs. references, and (iii) inferring latent drivers such as occupancy/exposure.

For time series prediction, an edge-based bi-directional LSTM predicts CO_2_, PM_2.5,_ and TVOC from a 14-sensor indoor system, reporting “high performance” but without numeric metrics in the abstract [73]. A second IoT study compares LSTM, SARIMA, and linear regression to forecast IAQL and gas levels 7 days ahead, using 90 days of history. It states an accuracy over 95% for each parameter (O_3_, PM, CO, CO_2_, TVOC, and temperature/RH) but does not specify RMSE/MAE [19]. For near-future pollution or biological particles, an LSTM estimates real-time and less than 60 min ahead concentrations of bioaerosols plus PM_2.5_/PM_10_ in an office and mall. Reported prediction accuracy is approximately 60–80% for bioaerosols and 90% for PM on test/time series sets [24].

For occupancy inference and IAQ management, RF and ANN map CO_2_ and ventilation operation to occupant count, achieving accuracies of 0.9102 (RF) and 0.9180 (ANN). Adding differential pressure reduced accuracy to 0.8916–0.8936 [21]. In public transport, classification or regression-style ML (LR, DT, RF, and XGBoost) predicts conditions tied to occupancy. XGBoost reached 91.25% accuracy for corridor data (CO_2_/PM) [27].

A large share addresses low-cost sensor calibration with linear or ensemble models. Linear calibration yields R^2^ > 0.8 with low errors, and Kalman filtering denoises O_3_ [74]. Calibration comparisons report CO at R^2^ of 0.918 (MLR), 0.912 (RF), and 0.924 (ANN), and NO_2_ at R^2^ of 0.890 (MLR), 0.697 (RF), and 0.809 (ANN) [36]. A Plantower PMS5003 evaluation shows calibration improvements in RMSE (up to 64%), mean normalized bias (MNB) up to 70%, and coefficient of variation (CV) over 50% across log-linear, non-log-linear, and RF [75]. Consumer sensor validation finds PM_2.5_ at R^2^ of 0.79, RMSE of 5.8 µg/m^3^, ρ of 0.69, limits of agreement width 30.1 µg/m^3^, and improved performance when averaging 5–30 min intervals [76]. Methodologically, formaldehyde calibration highlights that OLS is invalid under autocorrelation [77]. Optimized Gaussian Process Regression predicts indoor CO_2_ with R^2^ of 0.98874, RMSE of 4.20068 ppm, MAE of 3.35098 ppm, Nash–Sutcliffe of 0.9817, a20-index of 1 using real-time office features (occupants, area/person, outdoor temperature and wind, RH, and AQI) [78].

The accuracy analyzed in Table 5 comes from empirical ML validations from studies and not from manufacturer datasheets.

Improvements for reducing RMSE by up to 64% for PM sensors show post-calibration performance [75]. Temperature and RH are sources of errors for MOS and optical sensors [33,76].

Benchmarking studies indicate that LSTM achieves lower RMSE errors than RF for time series with long-term dependencies [79]. For example, Jarif et al. [80] report an RMSE of 3.699 for LSTM compared to 5.323 for RF for PM_2.5_ prediction on an hourly dataset from East Asia.

In the context of the present work, the objective was not to re-benchmark the algorithms, but to evaluate their suitability to the specific characteristics of public datasets. A complete ablation analysis on all dataset–algorithm combinations exceeds the scope of this synthesis study.

The correlation between the uncertainty profile of the sensors and the selection of algorithms is supported by recent ML-based calibration studies, as shown in Table 6. High-noise sensors are characterized by RF-specific decision aggregation, XGBoost, or LSTM-type temporal modeling. Calibrated sensors use linear or physical–statistical models.

Table 6 presents the sensor–algorithm correlation discussion to cite empirical studies that validate the mapping between sensor uncertainty profiles and the algorithm. The cited literature provides the evidence base for our selection guidelines.

A detailed and reference description for the present work is represented by the technical report of the European Commission [84]. The European Union Air Quality Directive indicates that measurement uncertainty should be the main indicator used for data evaluation. This paper analyzes the performance of low-cost sensors for air quality monitoring, comparing over 1400 laboratory and field tests. It evaluates accuracy, calibration, data transparency, sensor quality, and costs. For this reason, the authors of this paper recommend consulting the work [84] for specific details on outdoor air quality. The sensors used in evaluating outdoor air quality can also be integrated into the context of IAQ.

## 8. Discussion

The synthesis of the search results on the WoS platform shows the convergence between the evolution of ML models and the specifics of the IAQ issue. A quantitative literature analysis based on WoS queries for the period 2020-2025 presents a picture of the methodological interpretations of the performance reported in these works and the constraints imposed by the available data and sensors that enable data acquisition. This section will discuss the results obtained in this analysis.

The initial WoS analysis indicates six model families that excel at identifying IAQ. For linear regression, 132 articles were obtained, for LR 91, RF 77, LSTM 77, PCA 63, and Elastic Net 51. These values do not highlight the performance of the models, but their popularity among researchers. The large number of works on the linear model indicates that researchers prefer the model due to its simplicity of implementation and understanding. Linear regression estimates the direct effect of ventilation, occupancy, temperature, and RH on CO_2_, PM_2.5_, VOC concentrations, or other parameters involved in IAQ. Elastic Net is also a linear model, but it achieved 51 results. Multicollinearity within this model arises from multiple sensors acquiring variables that need to be correlated. In the case of this model, the values show that the IAQ datasets are wide with numerous redundant variables, and regularization becomes necessary for the stability of these models. LR guides IAQ applications toward classification. Essentially, applications integrated using the LR model indicate the acceptable or unacceptable state of the IAQ indicator, exceeding the threshold, the need for intervention, and the need for ventilation. RF and LSTM provided 77 articles, marking the transition toward the field of nonlinear and temporal models. RF is preferred because it is not sensitive to noise present in the data or to missing data resulting from sensor failure, battery discharge of the data acquisition equipment, power interruption, or other unforeseen incidents. LSTMs offer good results due to their ability to model repetitive daily cycles, sudden variations in pollutants, or other such specific IAQ behaviors. PCA offers 63 results. This is not a predictive model in itself, but rather a dimensionality reduction tool. It is usually integrated in combination with other methods, which is why it has yielded a large number of scientific papers on the WoS platform.

The performance value analysis is reported in terms of accuracy or R^2^. For IAQ classification tasks, most studies report accuracy over 90%, with maximum values of 99.37% for LSTM and 99.20% for RF. It should be mentioned that these values are obtained in rooms where the context is perfectly controlled. These values were obtained because the data were well-structured, processed, and labeled, allowing the models to separate the IAQ states. In the case of regression, the R^2^ values range between 82% and 98%, especially for CO_2_ and PM_2.5_ prediction. XGBoost models or hybrid RF-LSTM architectures achieve R^2^ values of up to 99%, which means a perfect fit on the test data. The authors of this paper recommend interpreting these performance indicators with caution, as they can sometimes come from intra-dataset evaluations, meaning the testing does not generalize to seasonal situations.

The analyzed public datasets vary in size, as well as in the number of parameters analyzed. The DALTON dataset contains approximately 89 million samples. Thanks to its large volume of recordings, it offers the possibility to explore multiple ML models, from the simplest to the most complex. Processing this dataset comes with the disadvantage of requiring a hardware-software infrastructure that allows the models to train on such a large volume of data. At the opposite end are datasets that aggregate records daily and are insufficient for sophisticated models, as is the case with DL models. The GAMS IAQ dataset contains 135,100 records, and the residential IoT sets have approximately 97,000 records. These are located in a compromise area regarding ML models.

The IAQ datasets used in this study exhibit variations in sampling frequency, types of sensors, missing values, and different calibrations. All these different characteristics derive from the research objectives for which these data were collected. Therefore, the diversity of collection protocols in the specialized literature makes the post-hoc recalibration of raw data a convoluted process, difficult to achieve for a direct comparative analysis between the quality of data sets. For these reasons, the authors of this article highlight the general objective of the dataset to guide readers in choosing a specific dataset for their research. The objective of the comparative analysis is not to assess the quality of these datasets, but to choose them based on the type of research conducted. A direct analysis based on a pipeline is impossible due to the lack of access to co-localized reference tools. A proposal for such a pipeline would involve the following steps:

- All series must be summarized to 5-min intervals using the arithmetic mean to standardize the resolution of the analysis;
- Eliminate values outside the plausible physical range (e.g., CO_2_: 350–5000 ppm; PM2.5: 0–500 µg/m^3^), according to EPA guidelines for low-cost sensors [85];
- Segments with >30% missing data in a 1-h window should be excluded;
- Gaps <5% can be treated through linear interpolation, a threshold justified by recent studies on imputation in IAQ data.

These conceptual pipeline stages are specific to any preliminary processing performed on a dataset and do not represent a unified calibration protocol that guarantees absolute comparability between studies.

Since there is no universal algorithm for IAQ, the authors of this article propose a selection guide based on two dimensions that correspond to the authors’ area of expertise:

1. Data size and granularity:

- For fewer than 10,000 records (e.g., School IAQ Milan and mono situ studies), linear models, RF, or GB are recommended. The authors recommend these models because they do not have a tendency to overfit.
- For a volume of 10,000–100,000 records (e.g., GAMS and IoT Residential), RF, XGBoost, and LSTM with 1–2 layer architectures are recommended because inter-seasonal validation is mandatory to evaluate generalization.
- For a number of over 100,000 records (e.g., DALTON), LSTM/GRU, CNN-1D, or hybrid architectures (RF-LSTM) are recommended as they can exploit long-term dependencies and reduce RMSE compared to static models.

2. Type of pollutant and dynamic behavior:

- CO_2_ has a slow dynamics, so linear models or RF are suitable for short-term prediction.
- PM_2.5_ or PM_10_ have high variability and dependence on RH/T, which is why RF or GB are recommended to compensate for the nonlinearities of low-cost sensors. LSTM is preferable when modeling seasonality or episodic events such as cooking or cleaning.
- VOC/TVOC fits with decision aggregation algorithms (RF and XGBoost) or LSTM with extended input windows (≥60 min) for noise filtering.
- Pollutants with episodic emissions (NO_2_ and CO) correlate with sequential models (LSTM/GRU) because they capture the delayed relationships between sources and concentrations.

**Figure 6:**
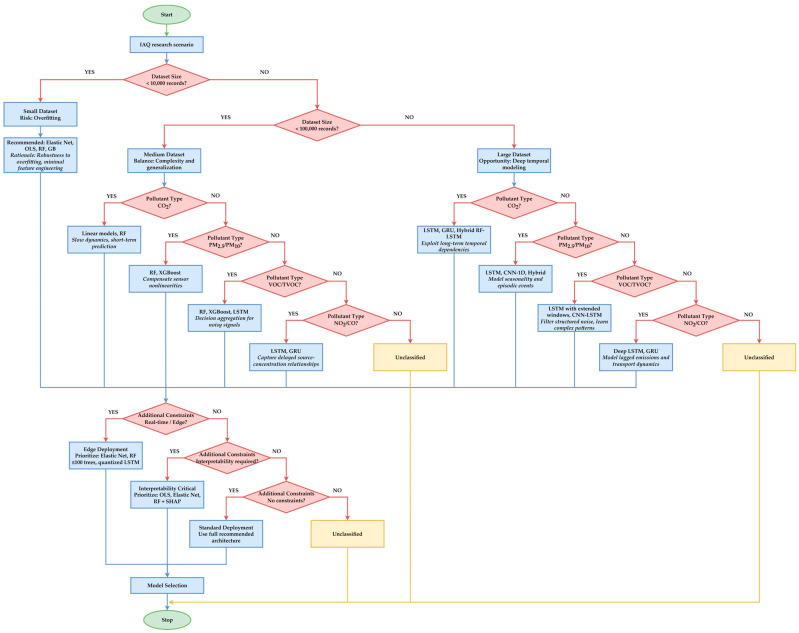
Flowchart for selecting algorithms based on the IAQ research scenario.

This selection guide is summarized in Figure 6.

This guide does not replace empirical validation. It represents a reproducible starting point for model selection. The authors’ proposal in Figure 6 reduces the risk of over- or under-training based on the specific constraints of the study. The framework prioritizes dataset size and pollutant dynamics as primary selection criteria. Secondary constraints (real-time deployment and interpretability requirements) may further refine the choice.

The distribution of articles for the types of sensors associated with IAQ parameters highlighted a very low number of articles. Therefore, the variations between 0 and 71 articles, representing a maximum number of results and corresponding to sensors that measure CO_2_, indicate the need for a larger volume of articles that investigate and discuss the hardware–software infrastructures related to the integration of these sensors for IAQ parameter acquisition. Restricting the analysis by combining ML models for sensors and IAQ context reduced the number of results to 26 for linear regression, 18 for RF, and 19 for LSTM. LR was reduced to only three studies. These results show that raw sensor data is predominantly used for regression and not for binary classification. From this analysis, the following ideas emerge:

1. There is no universally optimal model for IAQ prediction, and the choice depends on the purpose, data, context, and constraints.
2. Nonlinear and temporal models have superior performance, but the costs and complexity of training for generalization are also higher than those of linear models.
3. The quality, volume, structure, and acquisition method of datasets are the most important elements in the performance of ML algorithms.
4. The sensors used dictate the modeling problem, not the other way around.

This analysis is limited by the quality of reporting in the literature. The number of WoS articles reflects the low number of studies on the axes analyzed in this article. Therefore, future directions should include the development of standardized datasets spanning a large number of days, cross-site evaluation, the integration of physical methods with ML, the use of transfer learning, and the expansion of studies to less analyzed pollutants. Additionally, the authors of this paper also recommend reporting errors for ML models and their impact on health.

The selection of the model in IAQ studies is determined by methodological trade-offs between interpretability, the ability to capture nonlinearities, temporal dependence, processing time, integration difficulties, and data requirements. The main characteristics identified in the literature are:

• Linear regression distinguishes itself from other models in the context of IAQ through the simplicity of training, integration, interpretation, and estimation of the effects of environmental variables. A drawback of the model is that it cannot model nonlinear relationships or complex interactions, limiting it to contexts with simple dynamics or baseline establishment.
• Elastic Net solves the problem of multicollinearity in multi-sensor datasets and performs automatic variable selection. The disadvantage of these models is the need to adjust the regularization penalties.
• Logistic regression classifies IAQ states, such as labeling air quality as acceptable or unacceptable. The model’s performance decreases in the case of imbalanced distributions.
• RF and GB handle nonlinear relationships, noisy data, incomplete information, and inexplicable variations, and perform minimal preprocessing. These models are advantageous in practical situations that go beyond the barriers of theoretical study. The disadvantage of these models comes from the reduced transparency of the coefficients, which are approximated by various auxiliary algorithms. Another disadvantage arises from the predisposition to overfitting without inter-seasonal or cross-site validation.
• LSTM and DL architectures explicitly model temporal dependencies and daily cycles, being superior in short-term prediction. The disadvantage of these models arises from the need for large volumes of labeled data and, consequently, greater computational resources compared to training other models. Equally, these models have difficulties generalizing in untrained contexts.
• SVM is used for small to medium-sized datasets with nonlinear decision boundaries. And in the case of these models, the training cost increases with the volume of data.
• PCA is not a predictive model, but a dimensionality reduction and latent factor extraction tool. This is useful in preprocessing and denoising. The disadvantage of these models arises from the fact that information compression limits the physical interpretation of individual variables.

From the analysis conducted by the authors of this article, as well as from their experience in ML, there is no universally optimal algorithm for IAQ. The choice must be aligned with the modeling purpose, the structure of the dataset, the temporal granularity, the implementation constraints, and the context of its applicability.

The research findings indicate that ML provides analytical tools for IAQ, but success depends on careful alignment between data, models, purpose, and context. The analysis is aimed at researchers, engineers in the fields of structures, environment, software, IT professionals, AI-based content developers, decision-makers, and students, as it is necessary to clarify the current state of the field and guide future development in an informed manner.

The systematic search covered the WoS, Scopus, and IEEE Xplore databases for a quantitative analysis. Subsequently, for the methodological synthesis, the searches were narrowed to the WoS corpus to ensure a detailed analysis of the identified material’s content. This selection may exclude relevant studies published in journals indexed exclusively in Scopus or IEEE, but our article distinguishes itself from others in the literature through the analysis of the material’s content. The authors acknowledge a limitation introduced by restricting the content analysis to the WoS platform. This limitation is countered by the evaluated datasets (public and private), which predominantly originate from controlled or semi-controlled environments (offices, schools, residences, and nurseries) and are no longer dependent on the WoS platform. Ultimately, the performance of ML algorithms is excessively dependent on the quality, granularity, configuration, acquisition method, and preprocessing of the data. Based on the metric values reported in research articles, original contributions (R^2^ > 90%, accuracy > 90%) often reflect intra-dataset evaluations under stabilized conditions. Consequently, they do not guarantee that the models will perform equally well in cross-site scenarios, with noisy data, or with unlabeled sensor drift. Therefore, the direct comparability of the models is considered by the authors a limitation in the absence of standardized validation protocols and multi-site reference datasets. This shortcoming should be seen as a future research direction for those who will generate scientific materials in the field of IAQ.

## 9. Conclusions

The main objective of this literature review on IAQ is to identify ML algorithms that have been successfully used in prediction, regression, time series analysis, or other elements that would allow for their integration into everyday applications.

A primary result obtained in this research concerns researchers’ preference for linear models. For these reasons, OLS models, LR, and Elastic Net have generated a consistent volume of articles in the specialized literature. This is explained by the simplicity of understanding the algorithm, which allows the authors to make informed decisions. Because IAQ is a nonlinear process, the use of these models is limited. The reason IAQ is a nonlinear process is that complex interactions generated by episodic events such as cooking, cleaning, sudden changes in occupancy, variations in ventilation rate, and variations in outdoor air quality directly influence the value of IAQ. This behavior cannot be captured using linear models, which makes them incomplete. DT-based models like RF or XGB should be considered the best models analyzed in the current literature for applied IAQ research. These models report good performance for the regression of CO_2_, PM_2.5_, VOC concentrations, or classifications of IAQ states.

Another major contribution of this research is treating IAQ as a temporal process rather than a static problem. Specific time series models like LSTM, GRU, and CNN predict short-term pollutant concentrations or the IAQ index. The performance reported by these models confirms that architecture based on temporal dependencies, daily cycles, the inertia of ventilation systems, or other episodic elements directly influences the evolution of IAQ. The limitations of these models concern the need for large volumes of data, which also require accurate labeling. These are not always available in real-world contexts. This is where the need to investigate the datasets used in the literature also stems from.

A second drawback identified in the literature concerns the fact that some research reports very good results from analyzing a single room, which makes the model useless when applied in a different IAQ context. Datasets were identified as public or custom-made when acquired using proprietary sensors. The best dataset, named DALTON, was identified in the public datasets category. This includes data from multiple rooms and from various types of relationships formed by different human activities. Additionally, the other identified datasets are poorly inspected in the specialized literature. Consequently, it is recommended to investigate the possibility of training ML models using both public data and custom-made data, meaning data actually acquired through sensors.

The discussion regarding the metrics used shows a trend toward reporting accuracy and the R^2^ coefficient for evaluating algorithm performance. Most articles report exceptional values regarding the classification or approximation of the IAQ indicator value or other parameters involved in the process.

The results discussed in this research show that there is no universal algorithm optimized for IAQ. The method should be based on the application’s purpose, the variable to be predicted, the data structure, application-specific constraints, and data acquisition constraints.

The methodological directions identified in this article dedicated to IAQ can be extended to the field of ambient air quality. Both in indoor and outdoor environments, researchers face similar challenges in the context of ML. These are represented by the large volumes of data coming from sensor networks, the integration of multiple variables, handling missing values, calibrating low-cost sensors, detecting anomalies, and making short- or medium-term forecasts. From this perspective, the RF, XGBoost, LSTM, CNN, or hybrid models analyzed in this paper can be successfully adapted for monitoring outdoor atmospheric pollutants such as PM_2.5_, PM_10_, NO_2_, O_3_, or CO. However, the application of these methods in the outdoor environment also involves additional complexity, determined by the pronounced influence of weather conditions, spatial variability, hazard events, geographical specificity, and the regional transport of pollutants. For example, factors such as wind speed and direction, temperature, humidity, solar radiation, or local topography modify the dynamics of atmospheric pollutants and require the integration of additional variables into predictive models. Consequently, the experience accumulated in the field of IAQ represents a scientific starting point for the development of intelligent systems for monitoring and predicting ambient air quality.

The future directions recommended by the authors for investigation focus on the development of standardized datasets spanning several years, with high granularity, multiple room types, and a variety of activities carried out by occupants within these rooms. Additionally, the authors of this paper suggest that future research should expand the set of parameters, including both pollutant-type parameters and environmental measurements, to as many as possible.

## Figures and Tables

**Table 2:** Accuracy level reported in selected IAQ studies.

Research Objective	ML Algorithm	Accuracy (%)	Reference
IAQ classification and prediction	NN, LSTM	99.37	[17]
Ventilation operation prediction	RF	99.2	[18]
VOC/terpene detection & classification	RF	96	[25]
IAQ forecasting	LSTM, SARIMA	95	[19]
Occupancy detection	RF, XGBoost, DNN	95	[22]
PM_10_ prediction	IAQ-STL-ML (STL + Meta-learning)	94.93	[20]
HVAC + IAQ optimization	GRU, LSTM, CNN	92	[26]
Public transport IAQ prediction	XGBoost	91.25	[27]
Occupancy estimation (CO_2_-based)	RF, ANN	91.02	[21]
Bioaerosol prediction	LSTM	90	[24]
Radon risk mapping	CNN, LSTM	85	[23]

Note: ANN—Artificial neural network; DNN—Dense neural network; NN—Neural network; STL—Seasonal and trend decomposition using Loess.

**Table 3:** R^2^ level reported in selected IAQ studies.

Research Objective	Applicable Tasks	ML Algorithm	R^2^ (%)	Reference
IAQ parameter prediction	Hybrid physics-statistical modeling; parametric IAQ prediction with environmental dynamics constraints (PDE)	PDE–XGBoost	98.9	[28]
CO_2_ prediction (classroom)	Short-term CO_2_ forecasting; sequence modeling with temporal dependencies and daily cycles	RF–TPE–LSTM	98	[29]
Sensor calibration (CO_2_, PM_2.5_)	Low-cost sensor calibration; compensation of nonlinearities and drift under dynamic conditions	GB, kNN	97	[32]
PM_2.5_ calibration	Sequence-based calibration for PM sensors; capturing nonlinear RH/temperature dependencies	LSTM	96.2	[33]
Building performance and IAQ prediction	Robust nonlinear regression; prediction under noisy or incomplete data conditions	RF	95.78	[34]
IAQ prediction across rooms	Cross-zone spatial inference; multi-input/multi-output prediction for coupled variables	MLR, MLP, DT, RF, kNN	94	[35]
Gas sensor calibration (CO, NO_2_)	Gaseous sensor calibration; comparing linear vs. nonlinear models for drift compensation	MLR, RF, ANN	92.4	[36]
IAQI prediction	IAQI estimation; spatio-temporal causal modeling for air quality state prediction	ST-CCN-IAQI	91.7	[37]
IEQ dissatisfaction (PD%) prediction	Occupant perception prediction (PD%); regression for user-centered outcome variables	RF	91	[38]
Indoor CO_2_ prediction (no CO_2_ sensor)	Virtual sensing: inferring CO_2_ from BMS/meteorological variables without a dedicated sensor	RF	89	[39]
Indoor PM_2.5_ prediction	Regression for PM_2.5_ concentrations; handling episodic variations (cooking, cleaning)	RFR	84	[40]
Indoor O_3_ prediction	Reactive gas prediction; modeling nonlinear relationships with environmental factors	RF	83	[41]
HVAC IAQ modeling	Temporal modeling for HVAC control; short-term forecasting for proactive ventilation management	LSTM	82.1	[42]
Energy demand prediction (IEQ-driven)	Coupled energy-IAQ prediction; ensembles for interdependent variables	SVR + RF ensemble	82	[43]
Aerosol emission estimation	Episodic emission estimation; handling intermittent sources and noisy data	RF	73	[31]
Indoor PM_2.5_ prediction	Baseline linear regression (MLR) vs. nonlinear ensemble (RFR) for particulate matter; methodological benchmarking	RFR, MLR	72.2	[30]

Note: CCN—Causal Convolution Network; MLR—Multiple Linear Regression; PDE—Partial Differential Equations; RFR—Random Forest Regression; ST—Spatial–Temporal; TPE—Tree-Structured Parzen Estimator.

**Table 4:** Summary of the parameters and public datasets.

Dataset Name	Pollutants/IAQ Parameters Measured	Dataset Size (Number of Samples)	Data Quality Metrics	Sensor Models and Deployment Details	Reference
Indoor Air Quality Dataset with Activities of Daily Living in Low to Middle-income Communities	CO_2_, VOC, PM_1_, PM_2_._5_, PM_10_, NO_2_, C_2_H_5_OH, CO, temperature, RH, activity annotations (speech-to-text)	~89,100,000 samples (~13,646 h)	Binary validity flags, gaps ≤ 15 min interpolated via nearest-neighbor; gaps > 15 min filled with zeros; values rounded to 4 decimals; mean missing data: 11.82% (σ = 26.4%)	IoT sensing using ESP32-based devices; multi-modal sensing; 30 indoor locations across 4 Indian regions; 1–6 sensors per site; strategically placed at 1.0–1.5 m height (breathing zone); duration: 6 months; WiFi-based upload to cloud	[45,46]
Indoor Air Pollutants Using Low-Cost Sensors	PM_2_._5_ (µg/m^3^), NO_2_, NH_3_, CO (ppm), O_3_ (ppb), temperature (°C), RH (%), pressure (hPa)	173,468 records	ML-based calibration applied to low-cost sensor outputs to improve low-cost sensor accuracy	GP2Y1010AU0F (PM_2.5_), MiCS-6814 (NO_2_, NH_3_, CO), MQ131 (O_3_), and BME280 (temperature, RH, pressure); continuous indoor monitoring in Pune, India, over approximately 20 months	[49]
Extended Low-Cost IoT System Dataset	PM_2_._5_ (µg/m^3^), NO_2_, NH_3_, CO and CO_2_ (ppm), O_3_ (ppb), temperature (°C), RH (%), pressure (hPa)	139,448 records	ML-based calibration applied to low-cost sensor outputs; RMSE reduction of 55.42% reported for target system predictions	GP2Y1010AU0F (PM_2.5_), MiCS-6814 (NO_2_, NH_3_, CO), MQ131 (O_3_), CCS811 (CO_2_), and BME280 (temperature, RH, pressure); continuous indoor monitoring in Pune, India, over approximately 13 months	[50]
IoT Indoor Air Quality Dataset	PM_2_._5_ (µg/m^3^), PM_10_ (µg/m^3^), CO_2_ (ppm), TVOC (ppb), CO (ppm), temperature (°C), RH (%), light intensity (lux), motion (boolean), occupancy (count), ventilation status (Open/Closed)	97,458 records (18 February 2024–22 January 2025)	Measurements are recorded at 5-min intervals; multi-class classification (good, moderate, and poor); no missing data	Unspecified IAQ sensors; data from residential apartments (bedrooms, living rooms, and kitchens); data from 0.9–1.5 m height; varied household conditions: (cooking periods, occupancy levels, ventilation states)	[54]
GAMS Indoor Air Quality Dataset	PM_2_._5_ (µg/m^3^), PM_10_ (µg/m^3^), CO_2_ (ppm), VOC (mg/m^3^), temperature (°C), RH (%)	135,100 records (as of 7 January 2026)	Measurements are recorded at 1-min intervals using sensors with known accuracy (e.g., ±0.5 °C for temperature, ±40 ppm for CO_2_); it presents missing data and temporal gaps due to sensor failures	Unspecified IAQ sensors; data collected in Shanghai, Jingan district. China	[56]
School IAQ Dataset—Milan, Italy	PM_2_._5_ (µg/m^3^), PM_10_ (µg/m^3^), CO (ppm), CO_2_ (ppm), O_3_ (µg/m^3^, outdoor only), temperature (°C), RH (%), indoor pressure (hPa)	627 records (daily aggregated), from November 2023 to June 2024	Data are recorded at a 1-day interval per location; no explicit quality metrics or validation flags	Unspecified IAQ sensors; data collected from 3 school locations in Milan, Italy; 1.2–1.5 m height	[59]
Multi-Sensor Classroom IAQ Dataset	PM_1_ (µg/m^3^), PM_2_._5_ (µg/m^3^), PM_10_ (µg/m^3^), CO_2_ (ppm), VOC (µg/m^3^), temperature (°C), RH (%), dew point (°C), globe temperature (°C), air pressure (Pa), air velocity (m/s), occupancy, activity, ventilation	11,086 records (November 2021–June 2022)	Data are recorded at 1-min interval; 33 individual measurement sessions; sensor variability: all sensors combined: (37.35 ppm mean σ), max observed discrepancy (80 ppm)	Unspecified IAQ sensors; devices were placed at different positions within each room; data were measured from primary schools and university classrooms in Barcelona, Spain	[60]

**Table 5:** Typical accuracy ranges and ML calibration applicability for common IAQ sensors.

Sensor Type/Parameter	Typical Measurement Principle	Reported Accuracy/Error Range	ML Calibration Suitability	Reference
CO_2_	NDIR	Good stability, low cross-sensitivity vs. chemiresistive; specific ppm error not reported	High: LSTM for temporal drift compensation; GPR for multi-feature regression	[78]
PM_2.5_/PM_10_	Optical particle counter (laser scattering)	Calibration can improve RMSE up to 64%, MNB up to 70%; validated R^2^ = 0.79, RMSE = 5.8 µg/m^3^	High: LSTM for RH/temperature nonlinear compensation; RF for log-linear calibration	[33,75,76]
VOC/TVOC	MOS chemiresistive	Cross-sensitivity to RH/temperature; drift requires ML compensation	Medium-High: RF/SVM/XGBoost for compound detection; LSTM for sequence-based drift correction	[25]
NO_2_	Electrochemical/MO_x_	Calibration R^2^ = 0.890 (MLR), R^2^ = 0.697 (RF), R^2^ = 0.809 (ANN)	Medium: ANN/RF outperform linear baselines; requires environmental covariates	[36]
CO	Electrochemical (amperometric)	Calibration R^2^ = 0.918 (MLR), R^2^ = 0.912 (RF), R^2^ = 0.924 (ANN)	Medium: Ensemble methods improve robustness to interference	[36]
Temperature/RH	Thermistor/Capacitive MEMS	High accuracy, low cost; used as covariates for gas-sensor compensation; R^2^ = 0.94 (temperature), R^2^ = 0.94 (RH)	High: Standard inputs for calibration models (LSTM, RF, GPR)	[35]

Note GPR—Gaussian process regression.

**Table 6:** The correlation between the noise profile of sensors and the ML algorithms.

Sensor Profile (Uncertainty/Drift)	Typical Parameters/Technology	Recommended Algorithms	Noise Compensation Mechanism	Reference
High (>15% RMSE, rapid drift, cross-sensitivity to T/RH)	VOC/TVOC (MO_x_), NO_2_/O_3_ (electrochemical), PM (low-cost OPC)	LSTM/GRU, GB, RF	Decision aggregation (RF/GB) for outlier immunity; temporal windows (LSTM) for filtering structured noise; nonlinear compensation of temperature/RH interactions	[81]
Medium (5–15% RMSE, slow drift, relative stability)	CO_2_ (NDIR), temperature, RH (calibrated MEMS)	Elastic Net, RF, OLS	Approximately Gaussian residuals; regularization for multicollinearity; stable linear correlations	[82]
Low (<5% RMSE, reference or post-hoc calibrated)	CO_2_/PM (gravimetric/NDIR reference instruments)	Simplified regression, physico-statistical models	High precision; complexity shifts toward physical interpretation, energy optimization, or inter-site validation	[83]

## Data Availability

Not applicable.

## Supplementary Materials

The following supporting information can be downloaded at: https://www.mdpi.com/article/10.3390/s26092909/s1, Investigated_models.xlsx.

## Abbreviations

The following abbreviations are used in this manuscript:

1D CNN	One-Dimensional Convolutional Neural Network
AE	Autoencoder
ANN	Artificial Neural Network
ASHRAE	American Society of Heating, Refrigerating and Air-Conditioning Engineers
BMS	Building Management System
C_2_H_5_OH	Ethanol
CART	Classification and Regression Tree
CCN	Causal Convolution Network
CH_2_O	Formaldehyde
CNN	Convolutional Neural Network
CO	Carbon Monoxide
CO_2_	Carbon Dioxide
CV	Coefficient of Variation
DALTON	Indoor Air Quality Dataset with Activities of Daily Living in Low to Middle-income Communities
DL	Deep Learning
DNN	Dense Neural Network
DT	Decision Tree
DTR	Decision Tree Regression
EEA	European Environment Agency
EPA	U.S. Environmental Protection Agency
ETS	Error Trend Seasonal
GB	Gradient Boosting
GBDT	Gradient Boosting Decision Tree
GBM	Gradient Boosting Machine
GBR	Gradient Boosting Regression
GPR	Gaussian Process Regression
GRU	Gated Recurrent Unit
HAC	Hierarchical Clustering
HVAC	Heating, Ventilation, and Air Conditioning
IAQ	Indoor Air Quality
IAQI	Indoor Air Quality Index
IDW	Inverse Distance Weighting
IoT	Internet of Things
kNN	k-Nearest Neighbor
LiquidAdaptedNet	Liquid Neural Networks
LR	Logistic Regression
LSTM	Long Short-Term Memory
MAE	Mean Absolute Error
MAPE	Mean Absolute Percentage Error
MEMS	Integrated Micro-Electro-Mechanical Systems
ML	Machine Learning
MLP	Multilayer Perceptron
MLR	Multiple Linear Regression
MNB	Mean Normalized Bias
MOS	Metal Oxide Semiconductor
MO_x_	Metal-Oxide
MQTT	Message Queuing Telemetry Transport
MSE	Mean Squared Error
NDIR	Non-Dispersive InfraRed
NH_3_	Ammonia
NN	Neural Network
NO_2_	Nitrogen Dioxide
O_3_	Ozone
OLS	Ordinary Least Squares
OPC	Optical Particle Counters
PCA	Principal Component Analysis
PCR	Principal Components Regression
PD	Percentage Dissatisfied
PDE	Partial Differential Equations
PLS	Partial Least Squares
PM	Particulate Matter
RF	Random Forest
RFR	Random Forest Regression
RH	Relative Humidity
RMSE	Root Mean Squared Error
RNN	Recurrent Neural Network
RTD	Resistance Temperature Detector
SARIMA	Seasonal Autoregressive Integrated Moving Average
SHAP	SHapley Additive exPlanations
SO_2_	Sulfur Dioxide
ST	Spatial-Temporal
STL	Seasonal and Trend Decomposition using Loess
SVM	Support Vector Machine
SVR	Support Vector Regression
TPE	Tree-Structured Parzen Estimator
TVOC	Total Volatile Organic Compound
VOC	Volatile Organic Compound
WHO	World Health Organization
WoS	Web of Science
XGBoost	eXtreme Gradient Boosting
